# Comparative performance of scFv-based anti-BCMA CAR formats for improved T cell therapy in multiple myeloma

**DOI:** 10.1007/s00262-024-03688-4

**Published:** 2024-04-17

**Authors:** Sophia Stock, Luisa Fertig, Adrian Gottschlich, Janina Dörr, Florian Märkl, Lina Majed, Vivien D. Menkhoff, Ruth Grünmeier, Kai Rejeski, David M. Cordas dos Santos, Sebastian Theurich, Michael von Bergwelt-Baildon, Stefan Endres, Marion Subklewe, Sebastian Kobold

**Affiliations:** 1 Division of Clinical Pharmacology, Department of Medicine IV, LMU University Hospital, LMU Munich, Munich, Germany; 2 Department of Medicine III, LMU University Hospital, LMU Munich, Munich, Germany; 3 German Cancer Consortium (DKTK), Partner Site Munich, a partnership between the DKFZ Heidelberg and the LMU University Hospital, Munich, Germany; 4 Laboratory of Translational Cancer Immunology, LMU Gene Center, Munich, Germany; 5 Cancer- and Immunometabolism Research Group, LMU Gene Center, Munich, Germany; 6 Einheit für Klinische Pharmakologie (EKLiP), Helmholtz Zentrum München, German Research Center for Environmental Health (HMGU), Neuherberg, Germany

**Keywords:** Immunotherapy, Adoptive T cell therapy, T cell engineering, Chimeric antigen receptor, BCMA, Multiple myeloma

## Abstract

In multiple myeloma (MM), B cell maturation antigen (BCMA)-directed CAR T cells have emerged as a novel therapy with potential for long-term disease control. Anti-BCMA CAR T cells with a CD8-based transmembrane (TM) and CD137 (41BB) as intracellular costimulatory domain are in routine clinical use. As the CAR construct architecture can differentially impact performance and efficacy, the optimal construction of a BCMA-targeting CAR remains to be elucidated. Here, we hypothesized that varying the constituents of the CAR structure known to impact performance could shed light on how to improve established anti-BCMA CAR constructs. CD8TM.41BBIC-based anti-BCMA CAR vectors with either a long linker or a short linker between the light and heavy scFv chain, CD28TM.41BBIC-based and CD28TM.CD28IC-based anti-BCMA CAR vector systems were used in primary human T cells. MM cell lines were used as target cells. The short linker anti-BCMA CAR demonstrated higher cytokine production, whereas in vitro cytotoxicity, T cell differentiation upon activation and proliferation were superior for the CD28TM.CD28IC-based CAR. While CD28TM.CD28IC-based CAR T cells killed MM cells faster, the persistence of 41BBIC-based constructs was superior in vivo. While CD28 and 41BB costimulation come with different in vitro and in vivo advantages, this did not translate into a superior outcome for either tested model. In conclusion, this study showcases the need to study the influence of different CAR architectures based on an identical scFv individually. It indicates that current scFv-based anti-BCMA CAR with clinical utility may already be at their functional optimum regarding the known structural variations of the scFv linker.

**Supplementary Information:**

The online version contains supplementary material available at 10.1007/s00262-024-03688-4.

## Introduction

Multiple myeloma (MM) represents an incurable plasma cell neoplasm originating in the bone marrow. Almost all patients ultimately relapse with especially poor survival noted in patients with a high-risk cytogenetic profile or treatment-resistant disease [1]. Improved understanding of this disease has resulted in continuous evolution of treatment options leading to a new therapeutic repertoire [1]. Among them, chimeric antigen receptor (CAR) T cell therapy has emerged as a novel treatment modality with potential for long-term disease control. B cell maturation antigen (BCMA) is the most investigated target antigen for MM, mainly expressed by plasma cells and some mature B cells [2]. The U.S. Food and Drug Administration (FDA) approved the anti-BCMA CAR T cell products [3] *idecabtagene vicleucel (ide-cel)* [4] and *ciltacabtagene autoleucel (cilta-cel)* [5] for relapsed/refractory MM in 2021 and 2022, respectively. The first approved product *ide-cel* consists of a BCMA targeting single chain variable fragment (scFv), a CD8 alpha hinge/spacer and transmembrane domain (TM), a CD137 (41BB) intracellular costimulatory domain (IC or ICD), and a CD3zeta cytoplasmic domain (CD3ζ) [3, 4]. In terms of efficacy, only 33% of the patients treated with *ide-cel* exhibited a complete remission and 26% of all patients achieved MRD negativity [4]. ScFv-based anti-BCMA CAR T cell therapy remains a promising treatment approach, although coming with limitations including therapeutic resistance and toxicity [2, 4–6]. Besides the disease and patient’s characteristics, the CAR design influences its functionality [7–10]. The scFv, length and composition of the hinge/spacer, TM domain and ICD can impact CAR T cell functionality independently of each other [7, 11, 12].

It has been reported that a short amino acid linker (short linker) between the light (*V*_L_) and the heavy (*V*_H_) chain of the scFv part of 41BBIC-based anti-CD22 CAR T cells can lead to intermolecular CAR interactions that promote autonomous 41BB signaling, priming T cells for enhanced anti-tumoral effects [13]. In contrast, short linker versions of anti-CD33 and anti-CD19 CAR T cells did not exert such an effect [13]. This suggests that shortening the linker length of the scFv can induce CAR intermolecular pairing, but that this may vary between constructs and target antigens. It is thus promising to assess a short linker with a scFv-based BCMA-targeting CAR.

Another key aspect of CAR T cell functionality is the utilized costimulatory domain: CD28 is frequently employed both in preclinical models and in clinical products and leads to a more profound and rapid therapeutic impact, while 41BB (CD137) exhibits prolonged T cell persistence that is conducive to durable remissions [14]. However, this outcome is not uniformly observed across studies [15, 16], again indicating inter-CAR variability. Although CAR architecture targeting the same antigen can have a divergent impact on performance and efficacy, the optimal composition of such an scFv-based anti-BCMA CAR has not been investigated.

We aimed to perform a BCMA-target specific comparison of such CAR variations to evaluate the impact on anti-BCMA CAR T cell activity and to determine an optimized scFv-based anti-BCMA CAR design.

## Materials and methods

### Cell lines

MM cell lines RPMI 8226, U-266 (U266), JJN-3 (JJN3) and JK-6 (JK6) have been obtained from the German Collection of Microorganisms and Cell Cultures GmbH (DSMZ). Acute monocytic leukemia cell (AML) line THP-1 have been previously described [17]. All cell lines were cultured in a humidified incubator (37 °C, 5% CO_2_) and in RPMI 1640 medium containing 10–20% fetal bovine serum (FBS), 2 mM L-glutamine, 100 U/ml penicillin and 100 μg/ml streptomycin. All cell lines were lentivirally transduced with a pCDH-EF1a-eFly-eGFP plasmid [18]. After transduction, enhanced green fluorescent protein (eGFP)-positive cells were single cell sorted using a BD FACSAria III cell sorter, and expression of firefly luciferase (fLuc) was verified using a Bio-Glo luciferase assay system. Cells were frozen in 90% FBS with 10% DMSO and stored at − 80 °C or in liquid nitrogen for long-term storage. Cell lines were regularly checked for mycoplasma contamination. Authentication of cell lines by short tandem repeat (STR) profiling analysis was conducted in house.

### Generation of CAR constructs

All CAR constructs were second-generation CAR vector systems as previously described [17, 19–21] and either generated using conventional cloning techniques or codon-optimized and cloned into pMP71 retroviral vectors using commercial cloning services (Twist Bioscience, USA). c-Myc tags were used to detect CAR expression. Anti-BCMA CAR constructs were based on the patented sequence of the approved construct *idecabtagene vicleucel* [22]. CD8TM.41BBIC-based CAR vectors with either a short (referred as short linker CD8TM.41BBIC) or a long (referred as long linker CD8TM.41BBIC) amino acid linker in the extracellular antigen recognition domain between the *V*_L_ and *V*_H_ chain of the scFv section, CD28TM.41BBIC-based and CD28TM.CD28IC-based CAR vector systems were generated and used in primary human T cells. Besides the short linker construct, the other anti-BCMA CARs have the same long linker based on *ide-cel*. The long linker CD8TM.41BBIC therefore served as universal control. Anti-CD19 CAR T cells were designed based on anti-CD19-CAR-FMC63-28Z CAR T cells and used as negative controls [23], as previously described [17]. Single-cell clones were generated and screened for the highest level of virus production by determining T cell transduction efficiency, as previously described [20].

### CAR T cell generation and expansion

For virus production, retroviral pMP71 vectors carrying the CAR sequence (kindly provided by C. Baum, Hannover) were stably introduced into packaging cell lines 293Vec-Galv and 293Vec-RD114, as previously described [24]. T cells were isolated from peripheral blood mononuclear cells (PBMCs) of healthy donors by density gradient centrifugation, enriched by anti-CD3 microbeads (Miltenyi Biotec, Germany) and activated by Dynabeads human T-Activator CD3/CD28 (Life Technologies, Germany) before transduction. Human T cell transduction has been previously described [17, 19–21]. Untransduced T cells (UT) were isolated and activated like CAR T cells without transduction on day 2. T cells were expanded 1–2 weeks in human T cell medium (hTCM) containing RPMI 1640 with 2.5% human serum, 2 mM L-glutamine, 100 U/mL penicillin, 100 µg/mL streptomycin, 1% sodium pyruvate and 1% non-essential amino acid solution supplemented with recombinant human IL-2 (PeproTech, Germany/Novartis, Switzerland) used in a final concentration of 180 U/ml and IL-15 (PeproTech, Germany/Miltenyi Biotech, Germany) used in a final concentration of 2 ng/ml. Expansion steps were performed in the same manner for both untransduced and CAR T cells.

### T cell stimulation and killing assay using tumor cells

For co-culture experiments, 1.5 − 2.5 × 10^4^ tumor cells were plated in flat bottom 96-well plates. T cell numbers transduced with the indicated CAR constructs or untransduced T cells were added at different effector cell to target cell ratios (E:T ratios). Co-cultures were performed in hTCM without cytokines. Cytokines were washed out before the assay. Killing was assessed by luciferase-based killing assays using Bio-Glo™ Luciferase Assay System according to manufacturer’s protocol (Promega Corporation, USA).

### T cell stimulation assay using plate-bound recombinant protein

Flat bottom 96-well plates were coated overnight at 4 °C with Fc-tagged recombinant human BCMA (SinoBiological, China) with 2.0 µg/ml diluted in 50 µl PBS per well (0.1 µg/well). The next day, the protein-containing PBS was removed, plates were blocked with 70 µl 2% bovine serum albumin (BSA) dissolved in PBS for 30 min and washed after removal of the blocking solution with 100 µl PBS. After removal of the PBS serving as washing solution, 10^5^ T cells were resuspended in hTCM without cytokines and added into the protein-coated wells. Following an incubation time, culture supernatants were obtained for storage at  −  20 °C, and cells were used for flow cytometry-based analysis.

### Proliferation assay

For long-term co-culture experiments, 2.5 × 10^4^ U266 tumor cells were plated in flat bottom 96-well plates. T cell numbers transduced with the indicated CAR constructs or untransduced T cells (UT) were added at an E:T ratio of 2:1. Co-culture was performed in hTCM without cytokines for > 200 h. No further addiction or re-challenge of T cells with tumor cells was performed. At the end of cultivation time, proliferation and immunophenotype were assessed by flow cytometry and cytokine production was detected in culture supernatants by ELISA after storage at -20 °C.

### Cytokine protein level quantification

IFN-*γ*, interleukin (IL)-2 and granzyme B (Grzm B) release in co-culture supernatants was quantified by enzyme-linked immunosorbent assay (ELISA) according to manufacturer´s protocol (IFN-*γ* and IL-2: BD Biosciences, USA; Grzm B: R&D Systems, USA).

### Flow cytometry

For staining, cells were transferred into U-bottom 96-well plates and washed with ice-cold PBS and centrifuged (400 g, 5 min, 4 °C). Blocking of Fc receptors with TruStain FcX (BioLegend, USA) was performed (15 min). Dead cells were excluded after staining with a fixable viability dye (eFluor™ 780; eBioscience, USA). Surface proteins were stained (20 min, 4 °C). The following fluorophore-conjugated antibodies reactive to human antigens were used: anti-CCR7 (G043H7), anti-PD-1 (EH12.2H7), anti-CD8 (SK1), anti-TIM-3 (F38-2E2), anti-CD45RA (HI100), anti-CD3 (HIT3a or OKT3), anti-CD62L (DREG-56), anti-CD69 (FN50), anti-CD45 (HI30) and anti-CD4 (A161A1) (all from BioLegend). Anti-c-Myc (SH1-26E7.1.3) from Miltenyi Biotec was used for CAR detection. Target antigen expression was detected by BCMA-PE (19F2), CD19-PerCP-Cy5.5 (HIB19), and CD33-PE (P67.6). Quantification of absolute cell counts was carried out by using Count Bright™ Absolute Counting Beads (Thermo Fisher Scientific, USA). Cells were analyzed on a BD FACSCanto™ or BD LSRFortessa™ II flow cytometer, and data were analyzed with FlowJo software (version 10.7.2).

### Animal experiments

NOD.Cg-*Prkdc*^*scid*^* Il2rg*^*tm1Wjl*^/SzJ (NSG) mice were originally bought from Charles River Laboratories or Janvier or bred at the local facilities. Animals were housed in specific pathogen-free facilities and in groups of 2–5 animals per cage. Mice were held in facilities with a 12 h dark/12 h light cycle including a 30 min twilight phase at noise levels below 50 dB. Air velocity was held below 0.2 m/s. Air humidity in the facilities was between 45 and 60%, and the average temperature was held between 20 and 22 °C. We used 5–10 week-old female mice (*n* = 30) as recipients of matching appropriate tumor cell lines as described for each experiment. For bioluminescence imaging (BLI), mice were anesthetized using an isoflurane–oxygen mixture (1.5–2.5%) following intraperitoneal (i.p.) injection of BLI substrate (Xenolight D-Luciferin Potassium Salt, PerkinElmer, USA) into each mouse, according to the manufacturer’s protocol. An in vivo imaging system platform Lumina X5 (IVIS, PerkinElmer, USA) was used to measure BLI signal. The xenograft model using 2 × 10^6^ U266 tumor cells was established by i.v. injection. 2 × 10^6^ active CAR T cells were transferred at indicated times and numbers. Mice that had to be removed from animal experiments due to non-tumor-related toxicities (for example, did not have measurable BLI signal at the exclusion timepoint) were censored. Censored mice are indicated in the respective BLI images. All experiments were carried out randomized. For survival analyses, defined criteria (decrease in body weight or in general health condition) were taken as surrogates for survival and recorded in Kaplan–Meier plots. Percentage of tumor growth inhibition (TGI) was calculated with the formula [1 − (change of tumor volume in treatment group/change of tumor volume in control group)] × 100. Detailed experimental procedure can be found in the results section as a scheme before each experiment.

### Preparation of single-cell suspensions

Harvested organs and material of mice were processed to single-cell suspensions as previously described [20, 21]. Blood and bone marrow were diluted with PBS followed by erythrocyte lysis. Spleens were passed through 30 µm cell strainers, followed by erythrocyte lysis. Flow cytometry analysis is described above.

### Statistical analysis

At least three biological replicates for each experiment were analyzed unless otherwise indicated in the figure legends. Duplicates or triplicates for each group were performed. Two-tailed Student’s *t*-test was used for comparisons between two groups, while two-way ANOVA with Bonferroni posttest (multiple time points) or one-way ANOVA with Tukey’s posttest (single time points) was used for comparisons across multiple groups. A log-rank (Mantel-Cox) test was used to compare survival curves. Sample size was determined by *t*-test (two-tails) using the software G*Power 3.1 with given alpha, power, and effect size. All statistical tests were performed with GraphPad Prism 9 (GraphPad Software Inc., USA). *p*-values < 0.05 were considered statistically significant and represented as *p < 0.05, **p < 0.01, ***p < 0.001 and ****p < 0.0001. Graphs were designed with GraphPad Prism 9 (GraphPad Software Inc., USA) and Adobe Illustrator v26.0.2 (Adobe, USA). Where not otherwise indicated, results are presented as mean ± standard error of the mean (SEM).

## Results

### Comparative analysis of four anti-BCMA CAR constructs using different target cell lines

**Fig. 1:**
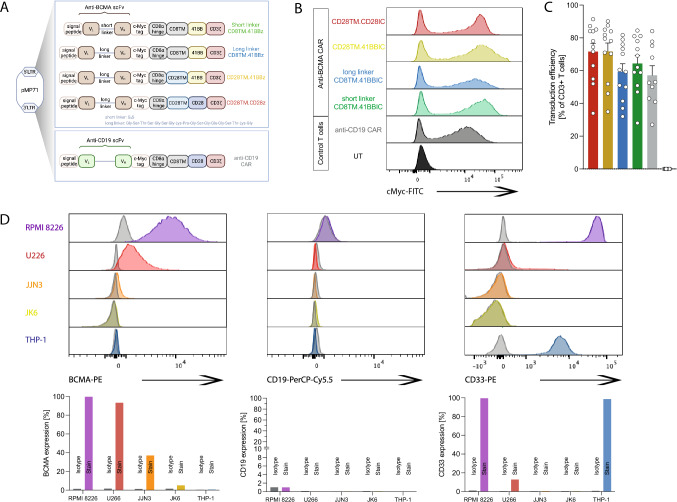
Evaluation of different anti-BCMA CAR constructs. **A** Schematic of CD28TM.CD28IC-based (red), CD28TM.41BBIC-based (yellow), and CD8TM.41BBIC-based anti-BCMA CAR constructs with either a long (blue) or short (green) linker between the light (*V*_L_) and the heavy (*V*_H_) chain of the scFv part. Anti-CD19 CAR T cells were used as negative control CAR T cells (gray). **B** + **C** Transduction efficiency in primary human T cells. Data for **B** one representative donor or **C** for *n* = 12 (anti-BCMA CAR) and *n* = 10 (anti-CD19 CAR) shown as means ± SEM. **D** Expression of human BCMA, CD19 and CD33 was detected on different multiple myeloma cells (RPMI 8226, U266, JJN3, JK6) and on the AML cell line THP-1. Expression was compared to isotype controls

In contrast to CD19-positive diseases, only 41BBIC-based anti-BCMA CAR products are approved (Suppl. Figure 1A) [3–5]. Here, we compared scFv-based BCMA-CAR constructs with either CD28 or CD8 as TM and 41BB or CD28 as ICD (Fig. 1A). We designed a novel anti-BCMA CAR with a short instead of a long linker between the *V*_L_ and *V*_H_ chain of the scFv (Fig. 1A). The long linker design is based on *ide-cel*; therefore, the long linker CD8TM.41BBIC anti-BCMA CAR was used as universal control. All constructs were successfully transduced (mean transduction efficiency > 60%) into primary human T cells (Fig. 1B + C). Primary myeloma cells vary in BCMA surface expression [25]. Therefore, several MM cell lines were used as target cell lines with a range of different BCMA, CD19 and CD33 expression profiles (Fig. 1D). While RPMI 8226 and U266 having the highest BCMA expression, for JJN3 and JK6 expression was low (Fig. 1D and Suppl. Figure 1B). All cell lines were CD19 negative so that an anti-CD19 CAR could be used as a negative control CAR for testing specificity (Fig. 1A-C). AML cell line THP-1 was used as an antigen-negative non-B cell or plasma cell-based control cell line (Fig. 1D and Suppl. Figure 1B).

### CD28 as costimulatory domain mediated a stronger activation then 41BB

**Fig. 2:**
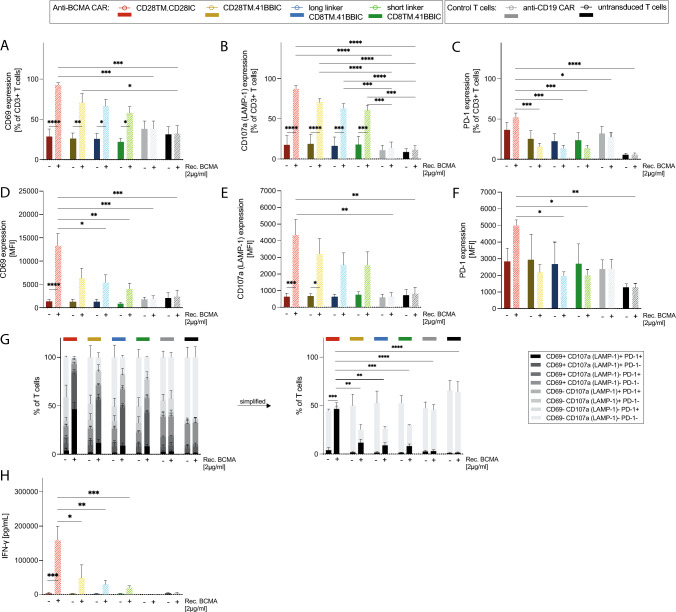
Specific CAR T cell activation by BCMA. **A** + **D** CD69, **B** + **E** CD107a (LAMP-1), **C** + **F** PD-1 expression as % of CD3+ T cells **A**-**C** and as MFI **D**-**F**, **G** CD69 ± CD107a (LAMP-1) ± PD-1 expression and **H** IFN-*γ* ELISA after stimulation of anti-BCMA CAR T cells (CD28TM.CD28IC: red; CD28TM.41BBIC: yellow; long linker CD8TM.41BBIC: blue; short linker CD8TM.41BBIC: green), anti-CD19 CAR T cells (gray) and untransduced T cells (black) with 2.0 µg/ml recombinant BCMA (*n* = 3 donors). Each experiment of subfigures **A**-**H** was performed in triplicates. Values in all graphs represent means ± SEM (* *p* < 0.05, ** *p* < 0.01, *** *p* < 0.001, **** *p* < 0.0001). Only selected p-values are shown. Statistical comparison was performed by a 2-way ANOVA with Bonferroni multiple comparison correction

All anti-BCMA constructs showed specific activation by recombinant BCMA after 24 h of stimulation, measured by expression of the T cell activation markers CD69 and CD107a (LAMP-1) as well as T cell exhaustion marker PD-1, which is also a marker of early T cell activation (Fig. 2A–F). Expression of CD69 with 93 ± 3% (Fig. 2A) and CD107a with 87 ± 4% (Fig. 2B) was the highest for CD28TM.CD28IC-based CAR T cells (Fig. 2A–B). PD-1 expression was relatively low compared to CD69 and CD107a (Fig. 2C). CD28TM.CD28IC-based CAR T cells also revealed a higher proportion of multifunctional (CD69 + CD107a + PD-1 +) cells with 46 ± 7% upon activation compared to 41BBIC-based anti-BCMA CAR constructs with between 8 and 12% (*p* < 0.01; Fig. 2G). Increased expression of T cell activation markers led to higher IFN-*γ* production of CD28TM.CD28IC-based CAR T cells (Fig. 2H) when stimulated with 2.0 µg/ml (*p* < 0.05) of recombinant BCMA. There were no differences between 41BBIC-CAR variants or scFv linker lengths (Fig. 2A-H).

### CD28TM.CD28IC-based CAR T cells showed higher in vitro cytotoxic capacity

**Fig. 3:**
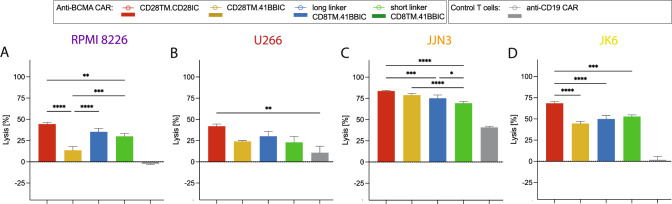
Highest cytotoxic capacity with CD28TM.CD28IC-based anti-BCMA CAR T cells.** A**-**D** Anti-BCMA CAR T cells (CD28TM.CD28IC: red; CD28TM.41BBIC: yellow; long linker CD8TM.41BBIC: blue; short linker CD8TM.41BBIC: green) were co-cultured with **A** RPMI 8226, **B** U266, **C** JJN3 or **D** JK6 tumor cell lines for 24 h at an indicated E:T ratio of 0.25:1. Anti-CD19 CAR T cells (gray) were used as negative controls, respectively. Cell lysis was quantified by luciferase-based killing assay. Subfigures show data of one representative donor out of three independent experiments. Each experiment was performed in triplicates. Values in all graphs represent means ± SEM (*p < 0.05, ** *p* < 0.01, *** *p* < 0.001, **** *p* < 0.0001). Only *p*-values for comparison of different anti-BCMA CAR constructs were shown except U266. Cytotoxic capacity of anti-BCMA CAR in comparison to anti-CD19 CAR T cells for RPMI 8226, JJN3 and JK6 were significant (*p* < 0.05). Statistical comparison was performed by a 2-way ANOVA with Bonferroni multiple comparison correction

We next compared the in vitro cytotoxic capacity. In general, all constructs were very potent against the used MM cells with an E:T ratio of 0.25:1 (Fig. 3A-D), particularly after 48 h and 72 h of co-culture reducing the window to perform comparative analysis (Suppl. Figure 2 + 3). CD28TM.CD28IC-based CAR T cells showed a higher cytotoxic capacity compared to the other constructs (Fig. 3A–D) particularly with RPMI 8226 (CD28TM.CD28IC vs CD28TM.41BBIC vs long linker CD8TM.41BBIC vs short linker CD8TM.41BBIC: 44 ± 2% vs 14 ± 5% vs 35 ± 4% vs 30 ± 3% for 24 h). BCMA low-expressing JJN3 and JK6 cell lines were killed similarly by the constructs and particularly JJN3 showed unspecific killing by anti-CD19 CAR and untransduced T cells due to higher intrinsic T cell sensitivity unrelated of targeting (Fig. 3 and Suppl. Figure 2). Similar effects were seen with E:T ratios of 2:1, 1:1, and 0.5:1 (Suppl. Figure 3). No clear differences were seen for the two linker lengths. As expected, AML cell line THP-1 could not be sufficiently killed (Suppl. Figure 4A).

### Shortening the scFv linker of 41BBIC-based anti-BCMA CAR increased cytokine production

**Fig. 4:**
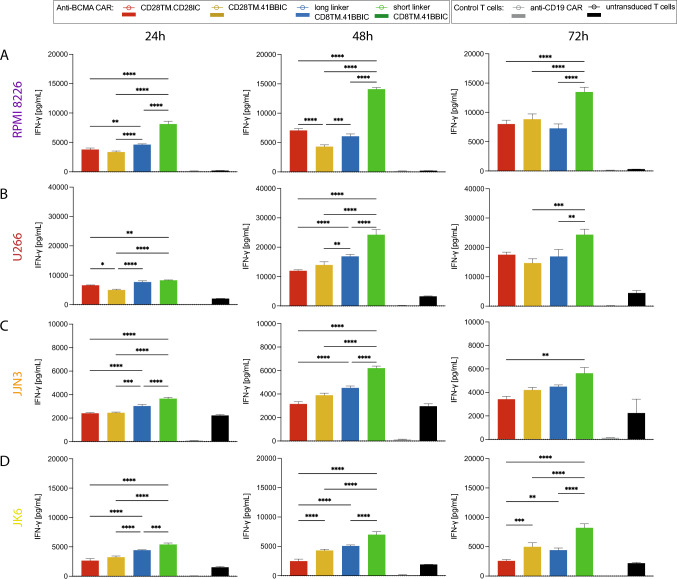
Highest IFN-*γ* production with short linker CD8TM.41BBIC-based anti-BCMA CAR. **A**-**D** IFN-*γ* ELISA with 24 h, 48 h, and 72 h co-culture supernatants of anti-BCMA CAR T cells (CD28TM.CD28IC: red; CD28TM.41BBIC: yellow; long linker CD8TM.41BBIC: blue; short linker CD8TM.41BBIC: green), anti-CD19 CAR T cells (gray) and untransduced T cells (black) with **A** RPMI 8226, **B** U266, **C** JJN3, or **D** JK6 in an E:T ratio of 2:1 for one representative donor out of three different donors. Each experiment was performed in triplicates. Values in all graphs represent means ± SEM (*p < 0.05, **p < 0.01, ***p < 0.001, ****p < 0.0001). Only *p*-values for comparison of different anti-BCMA CAR constructs were shown. Statistical comparison was performed by a 2-way ANOVA with Bonferroni multiple comparison correction

Shortening of the scFv linker led to an increased in vitro IFN-*γ* production (Fig. 4). After 72 h of co-culture in an E:T ratio of 2:1, short linker CAR T cells displayed a higher IFN-*γ* production compared to long linker versions in presence of RPMI 8226 (Fig. 4A), U266 (Fig. 4B), JJN3 (Fig. 4C) and JK6 (Fig. 4D). Donor variability led to less distinct effects when pooled data were analyzed (Suppl. Figure 5). Also, highest granzyme B and IL-2 production was seen with short linker CD8TM.41BBIC CAR T cells when co-cultured with RPMI 8226 for 72 h (Suppl. Figure 6A + B). Co-culture with THP-1 revealed a similar cytokine production as T cell only conditions (Suppl. Figure 4B-E).

### Long-term co-culture revealed higher proliferative capacity of CD28IC-based CAR T cells

**Fig. 5:**
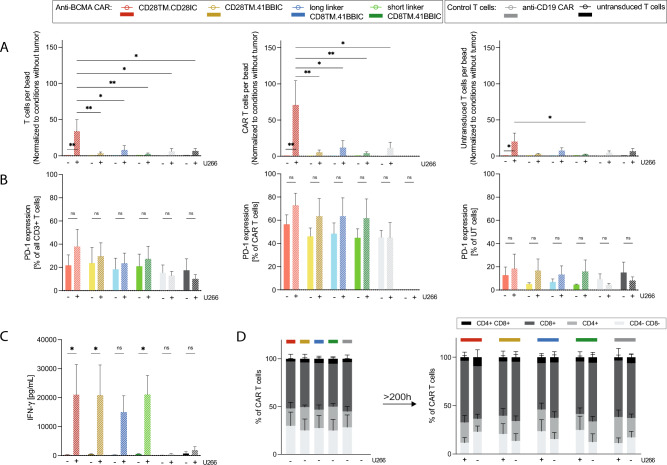
CD28TM.CD28IC-based anti-BCMA CAR T cells performed superior in long-term co-cultures. **A** Proliferative capacity, **B** PD-1 expression, **C** IFN-*γ* release and **D** T cell subtypes of anti-BCMA CAR T cells (CD28TM.CD28IC: red; CD28TM.41BBIC: yellow; long linker CD8TM.41BBIC: blue; short linker CD8TM.41BBIC: green), anti-CD19 CAR T cells (gray) and untransduced T cells (black) co-cultured for more than 200 h with U266 measured by FACS. **A** Proliferation is shown as T cells per bead, CAR T cells per bead or untransduced T cells per bead ratio and was normalized to conditions without tumor measured by FACS. Data is shown for three independent donors (*n* = 3). Each experiment was performed in triplicates. Values in all graphs represent means ± SEM (*p < 0.05, **p < 0.01, ***p < 0.001, ****p < 0.0001). Statistical comparison was performed by a 2-way ANOVA with Bonferroni multiple comparison correction

Previous reports have demonstrated that 41BBIC-based CAR T cells typically exhibit a longer persistence compared to CD28IC-based CAR T cells [14]. We assessed the impact of a long-term co-culture (> 200 h) with MM cells. Remarkably, the CD28TM.CD28IC-based anti-BCMA construct showed better proliferative capacity (*p* < 0.05) of all CD3 + T cells (33 ± 16) and CAR T cells (71 ± 34) compared to 41BBIC-based T cells (< 10) and CAR T cells (< 12) (Fig. 5A). No differences were seen of long and short linker versions (Fig. 5A). In contrast, the different CAR formats showed similar expression levels of PD-1 (Fig. 5B) and cytokine release (Fig. 5C). Analysis of T cell subtypes revealed no relevant changes during long-term co-culture (Fig. 5D).

### CD8TM.41BBIC CAR T cells had a higher proportion of central memory-like T cells

**Fig. 6:**
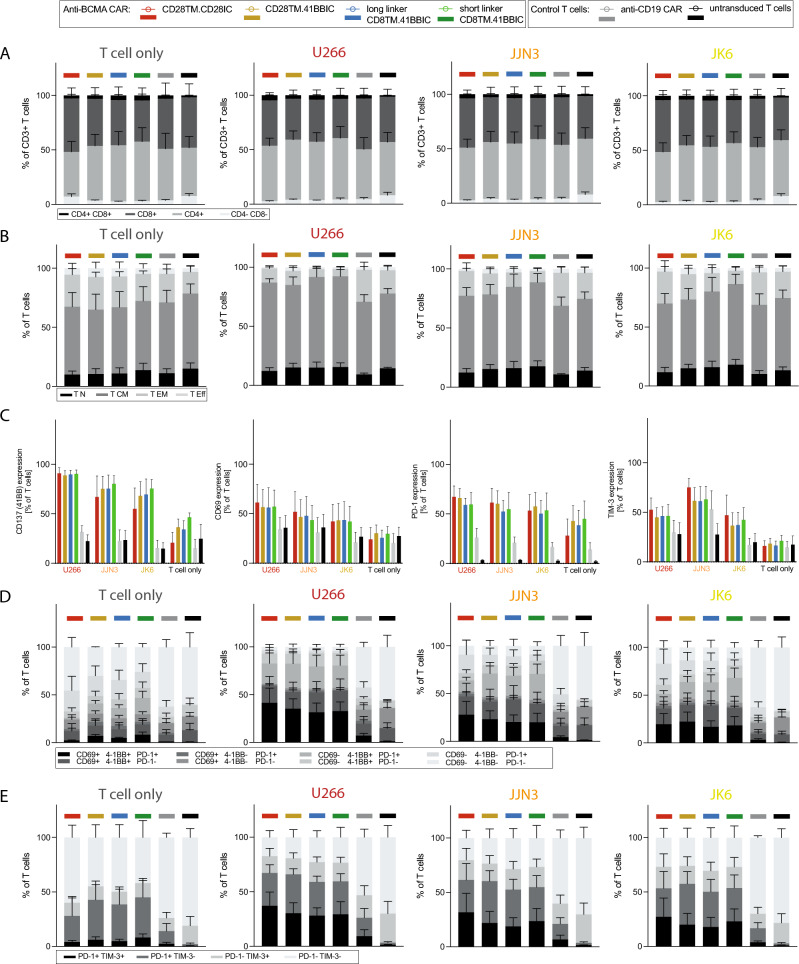
Phenotypic changes of CAR T cells after activation. Phenotype of anti-BCMA CAR T cells (CD28TM.CD28IC: red; CD28TM.41BBIC: yellow; long linker CD8TM.41BBIC: blue; short linker CD8TM.41BBIC: green), anti-CD19 CAR T cells (gray) and untransduced T cells (black) are shown after 48 h of co-culture in the presence of U266, JJN3 and JK6 or no tumor cells (T cell only conditions) of three donors (*n* = 3). **A** CD8+ and CD4+ T cell subtypes are shown. **B** T cell subpopulations for T cell differentiation status are shown. Naïve-like T (T_N_) cells were defined as CD45RA+CCR7+ , central memory-like T (T_CM_) cells as CD45RA-CCR7+ , effector memory-like T (T_EM_) cells as CD45RA-CCR7- and effector-like T (T_Eff_) cells as CD45RA+CCR7-. Changes in T cell subtypes and T cell subpopulations for anti-BCMA CAR T cells were not significant (ns). **C** Expression of 41BB (CD137), CD69, PD-1 and TIM-3 is shown. Only *p*-values for comparison of different anti-BCMA CAR constructs were shown. Comparison was not significant (ns). **D** Expression of CD69 ± 41BB ± PD-1 is shown. Changes for anti-BCMA CAR T cells were not significant (ns). **E** Expression of PD-1 ± TIM-3 is shown. Changes for anti-BCMA CAR T cells were not significant (ns). Each experiment was performed in triplicates. Values in all graphs represent means ± SEM (*p < 0.05, **p < 0.01, ***p < 0.001, ****p < 0.0001). Selected *p*-values are shown in the figures or described in the Results section. Statistical comparison was performed by a 2-way ANOVA with Bonferroni multiple comparison correction

We further assessed the phenotypic composition of the T cell product. For analysis of T cell subtypes, the CD8 to CD4 T cell ratio was similar for CD28IC-based and 41BBIC-based anti-BCMA CAR T cells after co-culture with MM cells (Fig. 6A). After co-culture with tumor cells, a trend towards a higher proportion of central memory-like T cells for CD8TM.41BBIC-based CAR T cells was seen compared to the CD28TM-based CAR T cells (Fig. 6B). Single marker analysis of activation and exhaustion marker expression was similar for all conditions (Fig. 6C), while the proportion of multifunctional T cells (CD69+ 41BB+ PD-1+) and exhausted T cells (PD-1+ TIM-3+) was highest for CD28TM.CD28IC CAR T cells, albeit without reaching statistical significance (Fig. 6D + E).

### Prolonged in vivo persistence and tumor control of CD28TM.41BBIC-based CAR T cells

**Fig. 7:**
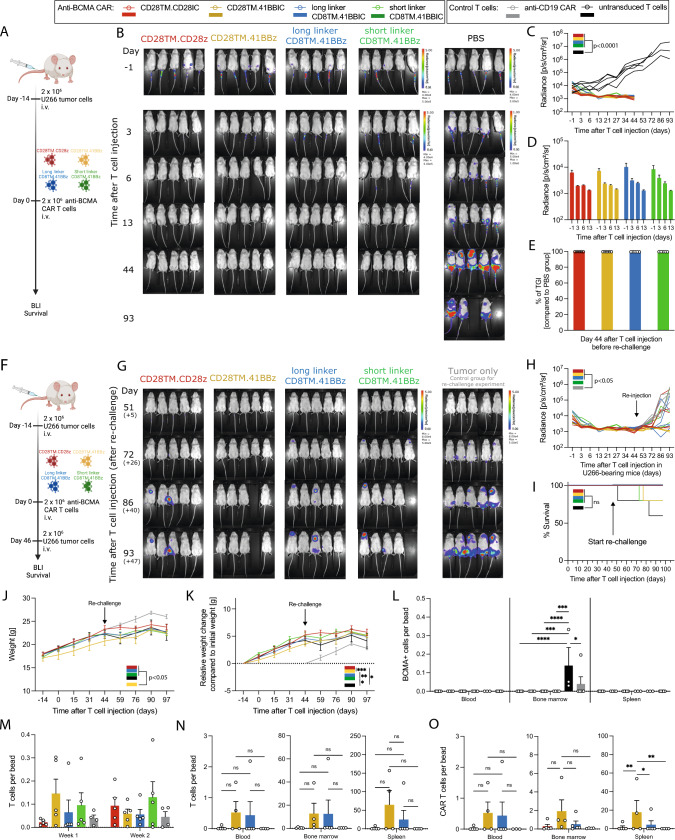
In vivo efficiency of anti-BCMA CAR T cells. **A** Experimental layout for **B**-**E**. Anti-BCMA CAR T cells (CD28TM.CD28IC: red; CD28TM.41BBIC: yellow; long linker CD8TM.41BBIC: blue; short linker CD8TM.41BBIC: green) or PBS (black) were intravenously (i.v.) injected in U266-bearing NSG mice. **B** BLI images, **C** + **D** quantification of tumor burden and **E** percentage of tumor growth inhibition (% of TGI) in U266 tumor-bearing mice after treatment with different CAR T cells (*n* = 5 mice per group). **F** Experimental layout for (**G**–**O**). **G** BLI images, **H** quantification of tumor burden, **I** survival curves and **J** + **K** body weight analysis of U266 bearing mice re-challenged 46 days after initial T cell injection with again tumor cells (*n* = 5 mice per group) and treatment naïve tumor only control mice (*n* = 5 mice per group). PBS-treated mice of **A**-**B** underwent no re-challenge. **L** BCMA + tumor cells per bead ratio for blood, bone marrow and spleen at time point of termination of the experiment on day 97 (+ 51), **M** T cells per bead ratio for blood in the first week and second week after CAR T cell injection. **N** T cells per bead ratio and **O** CAR T cells per bead ratio for cells detected in blood, bone marrow and spleen at time point of termination of the experiment. Values in all graphs represent means ± SEM (*p < 0.05, **p < 0.01, ***p < 0.001, ****p < 0.0001). Selected p-values are shown in the figures. Statistical comparison except for survival curves was performed by a 2-way ANOVA with Bonferroni multiple comparison correction. For Kaplan–Meier curves, statistical significance was calculated with log-rank test

To assess in vivo persistence and proliferation of anti-BCMA CAR T cell constructs, U266-bearing immunodeficient NSG mice were treated with 2 × 10^6^ active anti-BCMA CAR T cells or PBS (Fig. 7A). In the first days after treatment, CD28TM.CD28IC-based CAR T cells cleared the tumor slightly quicker than the 41BBIC-based CAR constructs (Fig. 7B-D). However, tumor clearance was comparable for all tested CAR constructs with a complete clearance of the tumor after 13 days compared to the PBS group (*p* < 0.0001) (Fig. 7B-D) and tumor growth inhibition (TGI) of nearly 100% (Fig. 7E).

To better evaluate T cell persistence, we re-challenged the anti-BCMA CAR T cell-treated survivor mice again with U266 cells on day 46 (+ 0) after initial T cell injection (Fig. 7F). Mice initially treated with PBS (black color) were not re-challenged (Fig. 7B, right column). As control mice for the re-challenge experiment (gray color), U266 tumor cells were injected into new NSG mice that had neither been treated with T cells nor PBS (Fig. 7G, right column). Remarkably, the CD28TM.41BBIC CAR T cells showed the best tumor control (Fig. 7G + H). Despite short linker CD8TM.41BBIC CAR T cells, anti-BCMA CAR T cell-treated groups showed better tumor control than the tumor only control group (gray; *p* < 0.05) in the re-challenge setting. No advantage in survival was seen as most of the mice were still alive at time point of termination (Fig. 7I). Weight analysis revealed (Fig. 7J + K) a significant reduction for CD28TM.41BBIC-based CAR T cells. At termination, BCMA+ tumor cells were only detectable in the bone marrow of the original PBS-treated mice (day 97) and the tumor only control mice for the re-challenge experiment (day 51 after re-challenge) (Fig. 7L). Bleeding of mice in the first and second week after T cell injection did not indicate differences for T cell detection (Fig. 7M). Upon termination of the experiment, detection of both T and CAR T cells in blood, bone marrow and spleen was only possible for the CD28TM.41BBIC and long linker CD8TM.41BBIC anti-BCMA CAR T cells (Fig. 7N + O), which was particularly evident for CAR T cells in the spleen (*p* < 0.05). In summary, shortening of the scFv linker did not lead to an advantage in an in vivo setting.

## Discussion

While commercial scFv-based anti-BCMA CAR T cell products have achieved excellent clinical results in pivotal studies, therapeutic failure and relapse are still observed [6]. CAR T cell therapy has evolved in recent years due to continuous improvements in CAR vector design [7, 8]. However, the optimal construction of an anti-BCMA CAR has yet to be defined. To evaluate the impact of CAR structure variations, we set out to perform a BCMA-target specific comparison on CAR T cell activity in vitro and in vivo so as to determine an optimized scFv-based anti-BCMA CAR design. We evaluated different scFv linker lengths and costimulatory domains.

In preclinical studies, anti-BCMA CAR T cell product *ide-cel* (bb2121) showed low antigen-independent signaling and potent in vitro killing of multiple myeloma tumor cells across a range of BCMA expression levels [26]. In comparison with studies testing CD28-based anti-BCMA CAR T cells, 41BB-based CAR T cells displayed improved persistence and a lower rate of toxicities including cytokine release syndrome (CRS) and immune effector cell-associated neurotoxicity syndrome (ICANS) [27]. Even though this and other clinical studies revealed promising results leading to approval of two commercial 41BB-based anti-BCMA CAR products, durable persistence of scFv-based anti-BCMA CAR T cells is generally poor and recurrence is common (for *ide-cel* median progression-free survival was 8.5 months and the median overall survival was 12.5 months in the real-world setting) [28]. Therefore, scFv-based anti-BCMA CAR constructs still require optimization.

Optimization of CAR design to enhance synthetic receptor-driven T cell functionality has been an important focus of the field [7–10]. Even though commercial CAR T cell products and preclinical CAR T designs share common features, all of them slightly differ from each other. Each part including the scFv, the hinge/spacer and TM domain and the ICD of the CAR can influence the functionality [11, 12]. While first-generation CAR T cells had only the CD3ζ chain, introduction of ICD particularly of CD28 and 41BB (CD137) markedly improved CAR T cell functionality [7]. CD28IC-containing CAR constructs were associated with enhanced levels of Th2-like cytokines like IL-4 and IL-10, and their kinetics of activation are more rapid than CAR vectors using 41BB as costimulatory domains [29–31]. Rapid activation of CD28IC-containing CAR T cells may make these cells more likely to initiate early-onset CRS in treated patients. Comparison of CAR constructs with identical scFvs in tumor treatment models revealed that 41BBIC-containing CAR T cells developed into more central memory-like T cells [14] with enhanced respiratory capacity, increased fatty acid oxidation, enhanced mitochondrial activity and better persistence compared to CD28IC-containing CAR T cells [14]. For now, two commercial 41BBIC-based anti-BCMA CAR products are approved. However, as persistence of these CAR T cells is generally poor [28], further improvement is required. We tested different combinations of CD28 and CD8 for TM domains as well as CD28 and 41BB for ICD for scFv-based anti-BCMA CAR constructs based on the design of the commercially available *ide-cel* product. In our study, CD28-based CAR T cells outperformed the 41BBIC-based CAR T cells regarding in vitro cytotoxicity, proliferative capacity, and overall activation status. Overall cytotoxicity after 48 and 72 h of co-culture was very high for all constructs. The long-term cytotoxic experiments cannot reasonably differentiate between the role of proliferation; therefore, proliferation might be a confounding factor regarding cytotoxicity. However, we observed better persistence of 41BBIC-based CAR T cells in vivo, as expected. These discrepancies may be model-related and therefore might be evaluated also with other cell lines in vivo or simply related to the utilized scFv as all constructs are based on the *ide-cel* scFv. By changing the scFv, the effect could be different due to a different ability for tonic signaling as well as avidity and affinity towards the target antigen.

Besides therapy-associated toxicities, another reason for leaking CAR T cell efficiency is tonic signaling by antigen-independent clustering of a CAR on the T cell [9, 10]. Multiple domains of the CAR have been associated with provoking or at least contributing to tonic signaling [10]. It can mediate spontaneous release of effector cytokines and the expression of surface markers associated with T cell exhaustion [10]. This led to poor in vivo activity and lack of in vivo function particularly when CD28 is used as ICD [10] due to distinct receptor biochemistry (not linker-driven clustering) of these CD28IC-based CARs [32, 33]. Replacement of a CD28 ICD with a 41BB ICD prevented the development of CAR T cell dysfunction suggesting a protective impact of tonic 41BBIC-based CAR signaling. Singh and colleagues could demonstrate that antigen-independent tonic signaling improved the function of 41BBIC-based anti-CD22 CAR T cells and can be enhanced by shortening the linker length between the *V*_L_ and *V*_H_ chain of the scFv part [13]. This shortening of the linker does not universally lead to clustering in every CAR product [13]. As such, it was recommended that each new CAR and target antigen should likely be evaluated individually for clustering and tonic signaling [13]. Thus, we evaluated the effect of the linker shortening in CD8TM.41BBIC-containing scFv-based anti-BCMA CAR T cells. We could not see a superior effector function of short linker CAR T cells except for a trend towards an increased in vitro cytokine production. This trend was not superior in a long-term co-culture setting measuring IFN-*γ*. The decay of the protein over time will underestimate the absolute amount produced. While decay will not be influenced by the CAR design, allowing direct comparison between constructs, it does not correct for cell numbers, nor for potential differences between cell populations in IFN-*γ* production. The findings of superior cytokine production in short-term co-cultures are in line with short linker anti-CD19 CAR T cells showing no tonic signaling with equivalent in vitro and in vivo cytotoxicity against CD19 + tumor cells but higher cytokine production compared to the long linker versions [13]. The superior short linker anti-CD22 CAR had a *V*_H_–*V*_L_ composition [13], while the here tested anti-BCMA CAR had a *V*_L_–*V*_H_ composition which may also influence the ability to perform intermolecular *V*_L_ and *V*_H_ pairing [34]. Each variable domain of the scFv contains three complementarity-determining regions (CDRs) forming loops and making up the unique antigen-binding site [35]. Additionally, four framework regions (FRs) in the scFv comprising beta-strands and additional loop regions are crucial for scFv stability [35]. The *V*_H_–*V*_L_ orientation is often favored because it leaves more distance between the scFv linker and the third CDR of the heavy chain, which usually is crucial for antigen-binding. However, *V*_L_–*V*_H_ orientation has been associated with increased expression and antigen-binding in some cases [36]. In other cases, similar surface expression and antigen-binding have been reported for either orientation [37]. Therefore, an ideal orientation of the *V*_L_ and *V*_H_ domains in scFvs still must be evaluated. The linker length itself heavily influences the oligomerization behavior of scFvs.

The scFv of most CARs is murine-derived and therefore can lead to host immunogenic responses and engineered T cell disappearance. Development of less immunogenic CAR constructs like CARs composed of camelid heavy-chain variable domains (VHH) has the potential to overcome this obstacle. Replacement of the targeting domain of a CAR with a single-chain antibody such as VHHs can also prevent tonic signaling as domain swapping and oligomerization is intrinsically not possible [38, 39]. Because VHH-based CARs are intrinsically not capable to oligomerize, shortening their linker length probably will not exert a major effect.

Another reason for the divergent results could be the target antigen itself as BCMA is a cell surface receptor and therefore has a different structure and function as CD19 and CD22, respectively. Since BCMA is primarily expressed on normal and malignant plasma cells and some mature B cells, it represents a suitable target for MM [40]. In this work, low BCMA expressing cell lines were also cleared by anti-BCMA CAR T cells. Primary myeloma cells vary in BCMA surface expression [25, 41]. Preclinical data of *ide-cel* (bb2121) revealed robust in vitro killing of MM cells independent of the BCMA expression levels or the presence of soluble BCMA [26, 27] also described by others [41]. Additionally, in the CARTITUDE-1 trial testing *cilta-cel* (NCT03548207), response rates were also independent of baseline BCMA expression [5]. Also, tumor cell killing is dictated by T cell and tumor cell intrinsic characteristics. In this specific setting, it seems that JJN3 and JK6 have a higher intrinsic T cell sensitivity unrelated to antigen targeting compared with RPMI 8226 or U266. We reasoned that these differences are not linked to BCMA nor BCMA targeting. A further promising target for MM is GPRC5D [42, 43]. Profound comparison of different CAR constructs designs and scFv linker lengths should also be performed with this clinically relevant target [42, 43]. It could be shown that structural alterations (biallelic loss of BCMA or GPRC5D) and antigen loss resulting from non-truncating missense point mutations in BCMA extracellular domain also mediate resistance to targeted immunotherapies [44].

Here, we used a c-Myc tag in the CAR construct to detect transduction efficiency. Antigen-based CAR expression is difficult to standardize and can be very CAR-dependent. Adding a c-Myc tag to the CAR sequence does not seem to negatively influence CAR performance in different CAR constructs [17, 19, 21]. Any minute alteration to a CAR sequence may potentially have functional consequences. The strategy was used by many in the meantime [45–47]. However, a systematic comparison on the CAR performance has not been performed to date.

In this present study, detailed analysis of in vitro and in vivo functionality of the anti-BCMA CAR constructs was performed; however, no further analysis concerning biophysical properties of CAR binding was conducted (i.e., affinity, avidity, and antigen density). Affinity and avidity can also substantially influence efficacy [48, 49]. It could be shown that moderate-affinity antigen-binding domain CAR have less off-target toxicity but were more effective [48]. The positive effects of short linker-mediated tonic signaling were reduced when using an affinity enhancement approach [13]. This could further help to understand the role of the CAR design and its interaction with the target antigen for CAR T cell effector function.

Our findings on anti-BCMA CAR T cells are not only relevant for MM but also for B cell NHL, as BCMA is also expressed in a relevant percentage of lymphoma samples [26]. It was shown that anti-BCMA CAR T cells do not react with normal PBMCs [26]. However, as many CD19, CD20 and/or CD22 targeting CAR T cell products are currently under investigation and four different anti-CD19 CAR T cell products are already approved, the role of BCMA as target antigen for the treatment of B cell lymphoma is for now less clear compared to CD19. Nonetheless, CD19/BCMA dual-targeting CAR approaches have been already tested for relapsed/refractory B cell NHL [50].

Anti-BCMA CAR are not functionally very sensitive to alterations in structure when BCMA-targeting scFv is conserved. While CD28 and 41BB (CD137) costimulation come with different in vitro and in vivo characteristics, we did not observe consistent domain-specific differences in terms of outcomes in the examined murine model. Shortening of the amino acid linker between the V_L_ and V_H_ chain of the scFv part of 41BBIC-based CAR is promising for CAR T cell therapy. However, the short scFv linker strategy could not relevantly improve the clinically approved scFv-based anti-BCMA CAR construct in this study. This may indicate that the scFv linker design of this CAR cannot be further improved by altering the length of the amino acid linker as proposed here and would require more elaborate modules such as cytokine signaling (so-called armored or fourth-generation CARs). This study showcases the need to study the influence of different CAR architectures based on an identical scFv individually. Optimizing anti-BCMA CAR constructs may improve outcomes; however, there are many variables that limit the success.

### Supplementary Information

Below is the link to the electronic supplementary material.

Supplementary file1 (PDF 3928 KB)

## Data Availability

All data needed to evaluate the conclusions in the paper are present in the paper and/or the supplementary files. Additional materials and data are available upon reasonable request.

## Abbreviations

BCMA: B cell maturation antigen
BLI: Bioluminescence imaging
BSA: Bovine serum albumin
CAR: Chimeric antigen receptor
CD3ζ: CD3zeta
CDR: Complementarity-determining region
CRS: Cytokine release syndrome
DMEM: Dulbecco's Modified Eagle's Medium
DSMZ: German Collection of Microorganisms and Cell Cultures GmbH
ECD: Extracellular domain
eGFP: Enhanced green fluorescent protein
E:T ratio: Effector cell to target cell ratio
FDA: U.S. Food and Drug Administration
FBS: Fetal bovine serum
FITC: Fluorescein isothiocyanate
fLuc: Firefly luciferase
FR: Framework region
Grzm B: Granzyme B
hTCM: Human T cell medium
ICANS: Immune effector cell-associated neurotoxicity syndrome
IC: Intracellular costimulatory domain
ICD: Intracellular costimulatory domain
IL: Interleukin
Ig: Immunoglobulin
i.v.: Intravenous
i.p.: Intraperitoneal
MM: Multiple myeloma
NHL: Non-Hodgkin lymphoma
NSG: NOD.Cg-*Prkdc*^*scid*^* Il2rg*^*tm1Wjl*^/Sz
PBMC: Peripheral blood mononuclear cell
PBS: Phosphate-buffered saline
ROB: Regierung von Oberbayern
RPMI: Roswell Park Memorial Institute
scFv: Single chain variable fragment
SEM: Standard error of the mean
STR: Short tandem repeat
T_CM_: Central memory-like T cell
T_Eff_: Effector-like T cell
T_EM_: Effector memory-like T cell
TGI: Tumor growth inhibition
T_H_: T helper cell
TLS: Tumor lysis syndrome
TM: Transmembrane domain
TME: Tumor microenvironment
T_N_: Naïve-like T cell
T_SCM_: Stem cell memory-like T cell
UT: Untransduced T cell
*V*_H_: Heavy chain of the scFv part
VHH: Camelid heavy-chain variable domains
*V*_L_: Light chain of the scFv part
