# Supplementary material for: Comparative performance of scFv-based anti-BCMA CAR formats for improved T cell therapy in multiple myeloma
Source: Cancer Immunol Immunother. 2024 Apr 17;73(6):100. doi: 10.1007/s00262-024-03688-4 (PMC11024081; doi:10.1007/s00262-024-03688-4)
Supplement: Supplementary file 1 — Supplementary file1 (PDF 3928 KB) [file 262_2024_3688_MOESM1_ESM.pdf]

SUPPLEMENTARY FIGURES

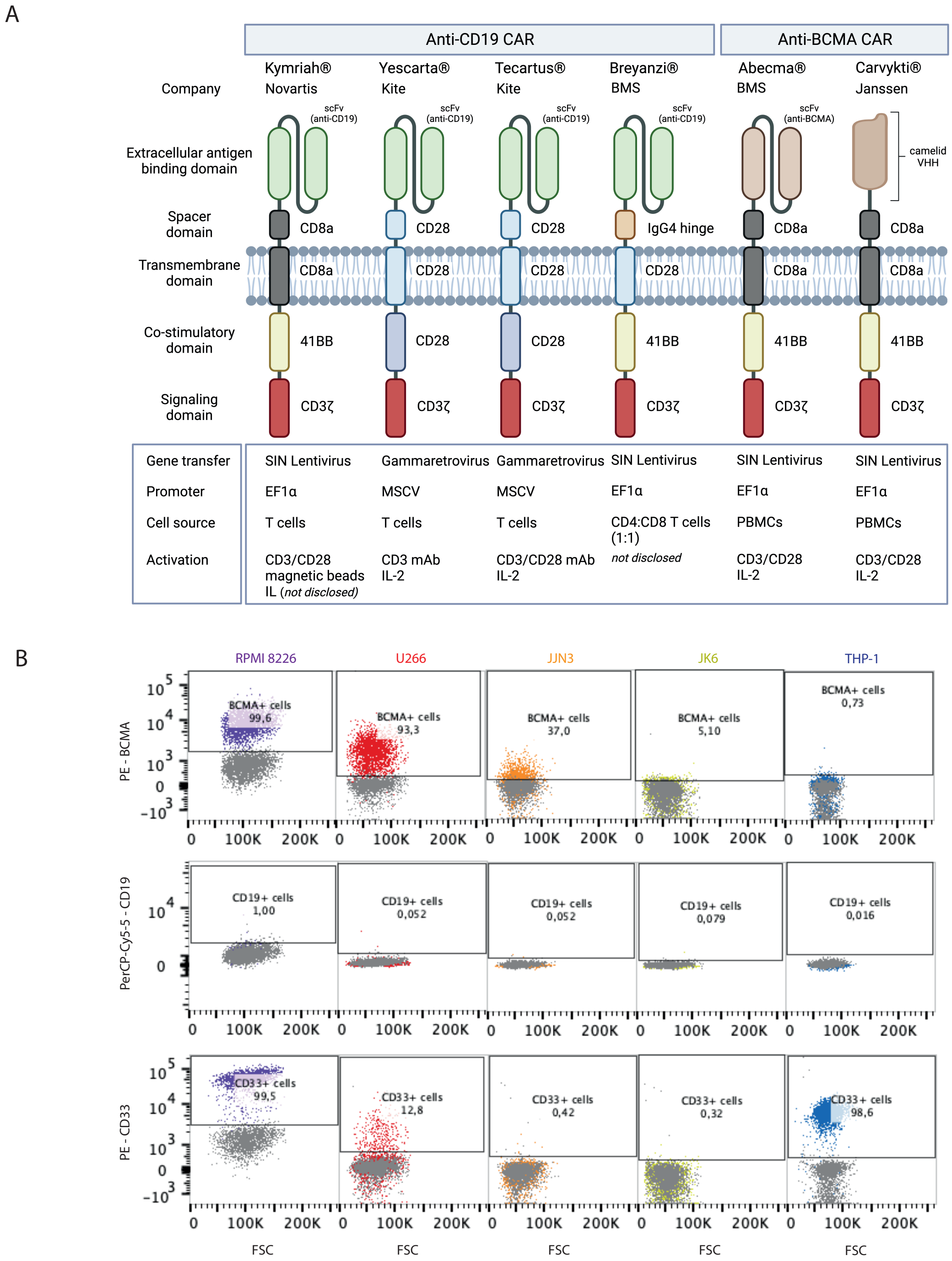

Suppl. Figure 1. FDA-approved CAR T cell products and surface marker expression on target cell lines. **A** Overview about the currently FDA-approved anti-CD19 and anti-BCMA CAR T cell products. **B** Expression of BCMA, CD19 and CD33 was detected on multiple myeloma cells (RPMI 8226, U266, JJN3, JK6) and on the AML cell line THP-1 and compared to isotype controls by flow cytometry.

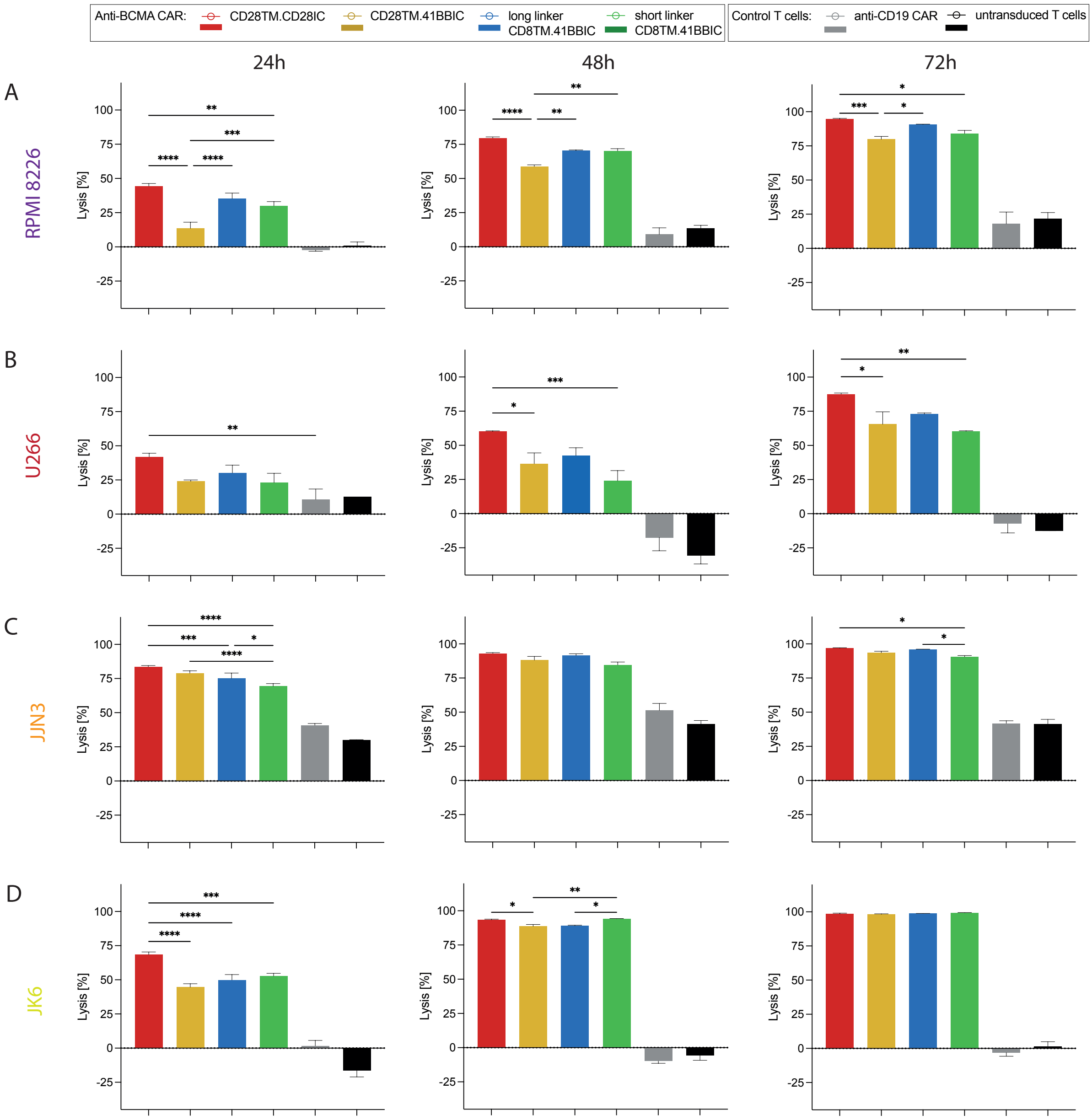

Suppl. Figure 2. Highest cytotoxic capacity with CD28TM.CD28IC-based anti-BCMA CAR T cells.
A-D Anti-BCMA CAR T cells (CD28TM.CD28IC: red; CD28TM.41BBIC: yellow; long linker CD8TM.41BBIC: blue; short linker CD8TM.41BBIC: green) were co-cultured with **A** RPMI 8226, **B** U266, **C** JJN3 or **D** JK6 tumor cell lines for 24 h, 48 h, and 72 h at an indicated E:T ratio of 0.25:1. Anti-CD19 CAR T cells (gray) and untransduced T cells (black) were used as negative controls, respectively. Cell lysis was quantified by luciferase-based killing assay. Subfigures show data of one representative donor out of three independent experiments. Each experiment was performed in duplicates or triplicates. Values in all graphs represent means ± SEM (* $p < 0.05$, ** $p < 0.01$, *** $p < 0.001$, **** $p < 0.0001$). Only p-values for comparison of different anti-BCMA CAR constructs were shown except U266 24 h. Statistical comparison was performed by a 2-way ANOVA with Bonferroni multiple comparison correction.

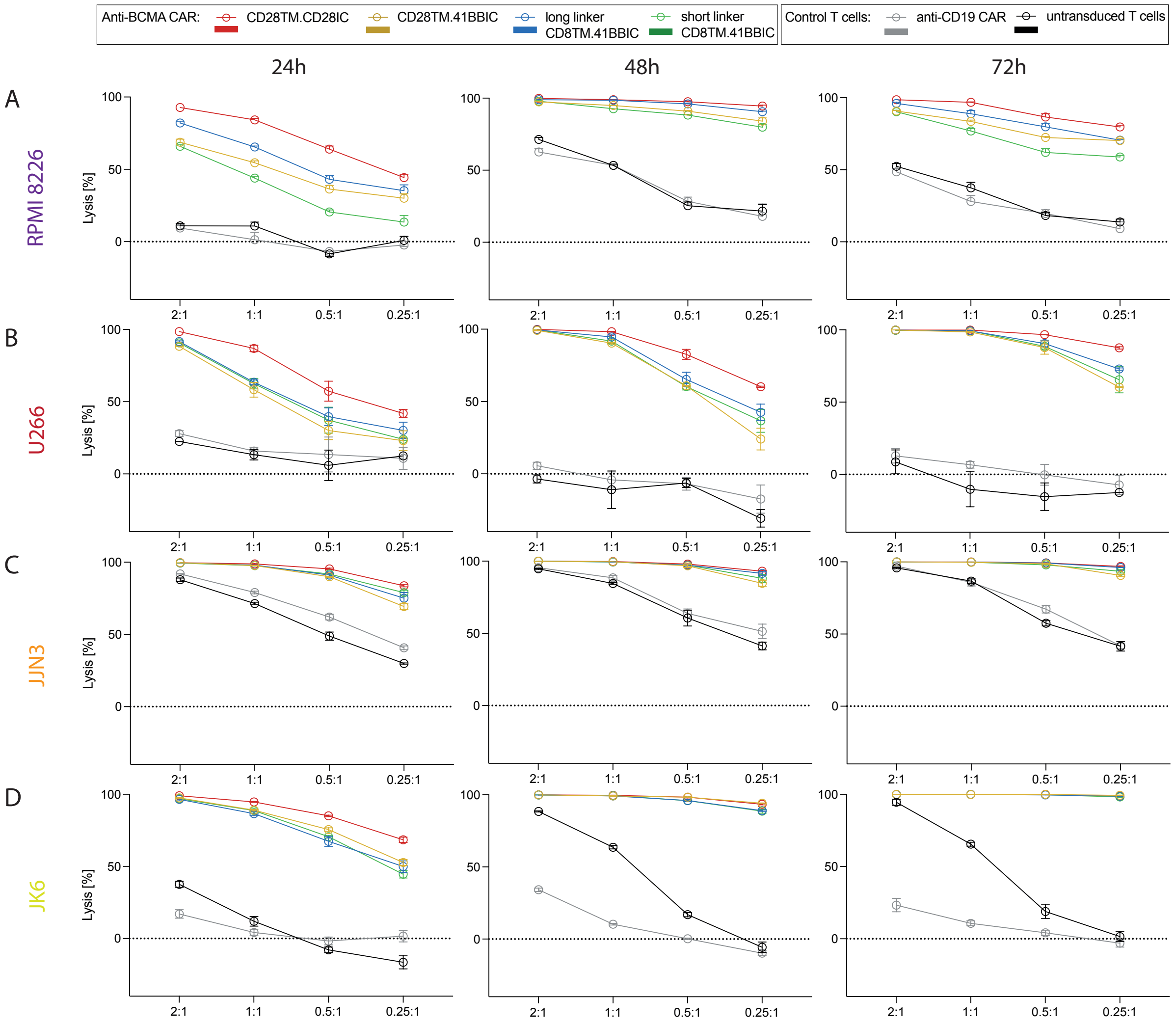

Suppl. Figure 3. Highest cytotoxic capacity with CD28TM.CD28IC-based anti-BCMA CAR T cells.
A-D Anti-BCMA CAR T cells (CD28TM.CD28IC: red; CD28TM.41BBIC: yellow; long linker CD8TM.41BBIC: blue; short linker CD8TM.41BBIC: green) were co-cultured with **A** RPMI 8226, **B** U266, **C** JJN3 or **D** JK6 tumor cell lines for 24 h, 48 h, and 72 h at indicated E:T ratios of 2:1, 1:1, 0.5:1 and 0.25:1. Anti-CD19 CAR T cells (gray) and untransduced T cells (black) were used as negative controls, respectively. Cell lysis was quantified by luciferase-based killing assay. Subfigures show data of one representative donor out of three independent experiments. Each experiment was performed in triplicates. The p-values are not integrated due to clarity of the figure.

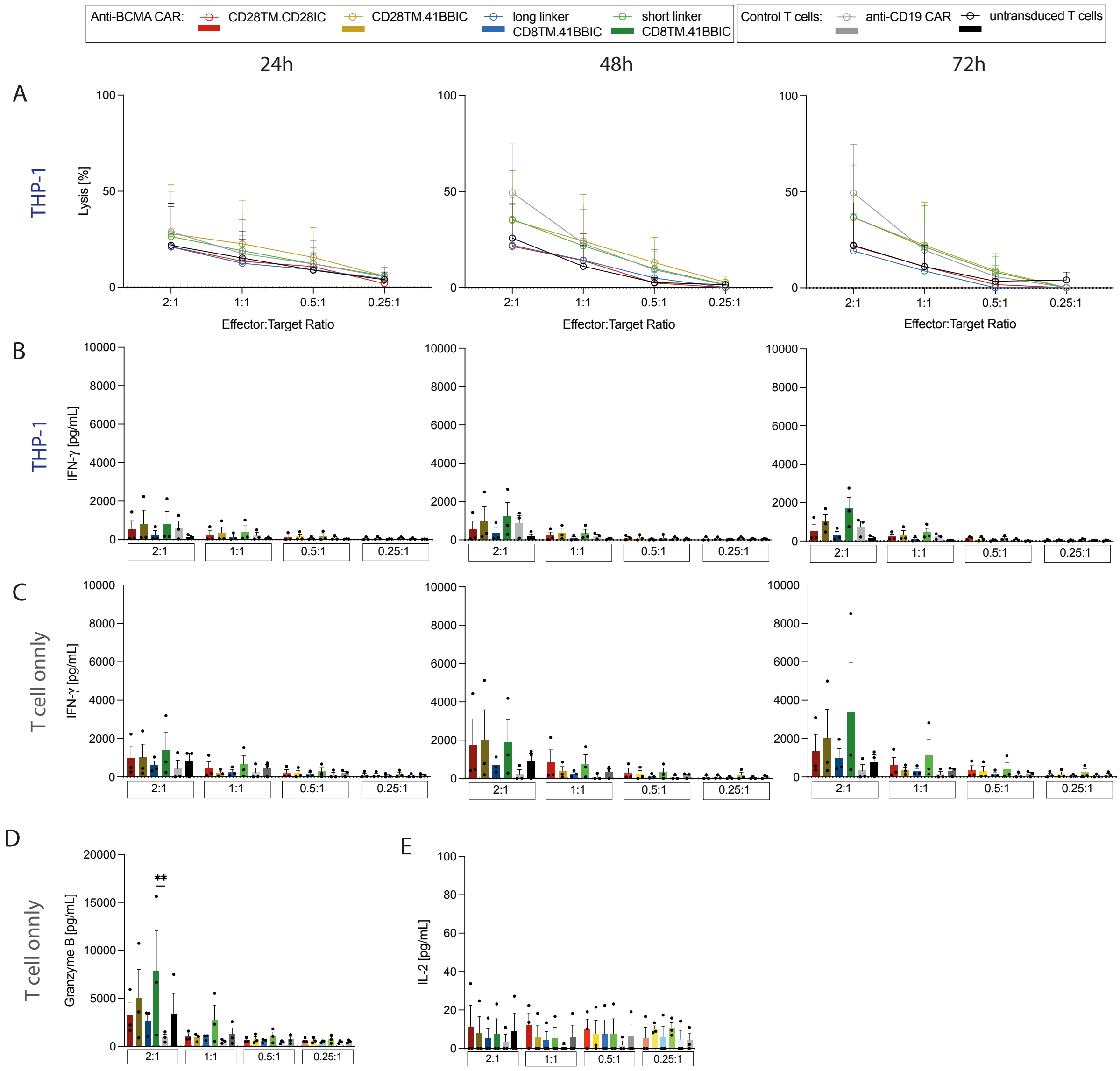

Suppl. Figure 4. Anti-BCMA CAR T cell functionality is specific for BCMA.

A Anti-BCMA CAR T cells (CD28TM.CD28IC: red; CD28TM.41BBIC: yellow; long linker CD8TM.41BBIC: blue; short linker CD8TM.41BBIC: green) were co-cultured with BCMA-negative THP-1 tumor cells for 24 h, 48 h, and 72 h at the indicated E:T ratios. Anti-CD19 CAR T cells (gray) and untransduced T cells (black) were used as controls, respectively. Cell lysis was quantified by BLI. Subfigures show pooled data of three different donors (n=3). **B-C** IFN-γ ELISA with 24 h, 48 h, and 72 h co-culture supernatants, **D** Granzyme B and **E** IL-2 ELISA with 72 h co-culture supernatants of anti-BCMA CAR T cells (CD28TM.CD28IC: red; CD28TM.41BBIC: yellow; long linker CD8TM.41BBIC: blue; short linker CD8TM.41BBIC: green), anti-CD19 CAR T cells (gray) and untransduced T cells (black) with **B** THP-1 or **C-E** without tumor cells for three independent donors (n=3). Each experiment was performed in triplicates. Values in all graphs represent means ± SEM (* $p < 0.05$, ** $p < 0.01$, *** $p < 0.001$, **** $p < 0.0001$). Only selected p-values are shown. Statistical comparison was performed by a 2-way ANOVA with Bonferroni multiple comparison correction.

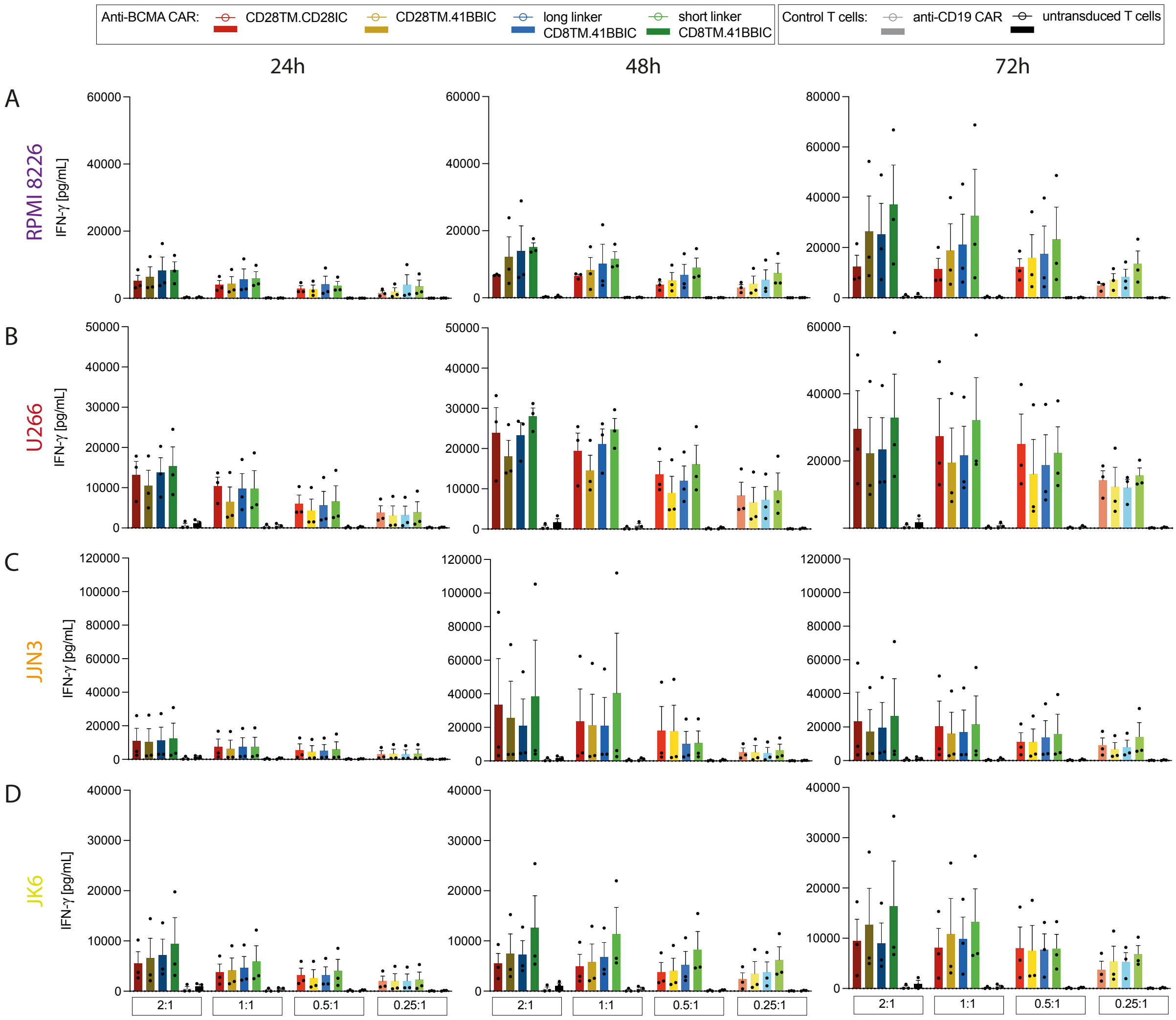

Suppl. Figure 5. Highest IFN-γ production with short linker CD8TM.41BBIC-based anti-BCMA CAR. **A-D** IFN-γ ELISA with 24 h, 48 h, and 72 h co-culture supernatants of anti-BCMA CAR T cells (CD28TM.CD28IC: red; CD28TM.41BBIC: yellow; long linker CD8TM.41BBIC: blue; short linker CD8TM.41BBIC: green), anti-CD19 CAR T cells (gray) and untransduced T cells (black) with **A** RPMI 8226, **B** U266, **C** JJN3, or **D** JK6, for three independent donors (n=3). Each experiment was performed in triplicates. Values in all graphs represent means ± SEM (* $p < 0.05$, ** $p < 0.01$, *** $p < 0.001$, **** $p < 0.0001$). Only p-values for comparison of different anti-BCMA CAR constructs were shown. Statistical comparison was performed by a 2-way ANOVA with Bonferroni multiple comparison correction.

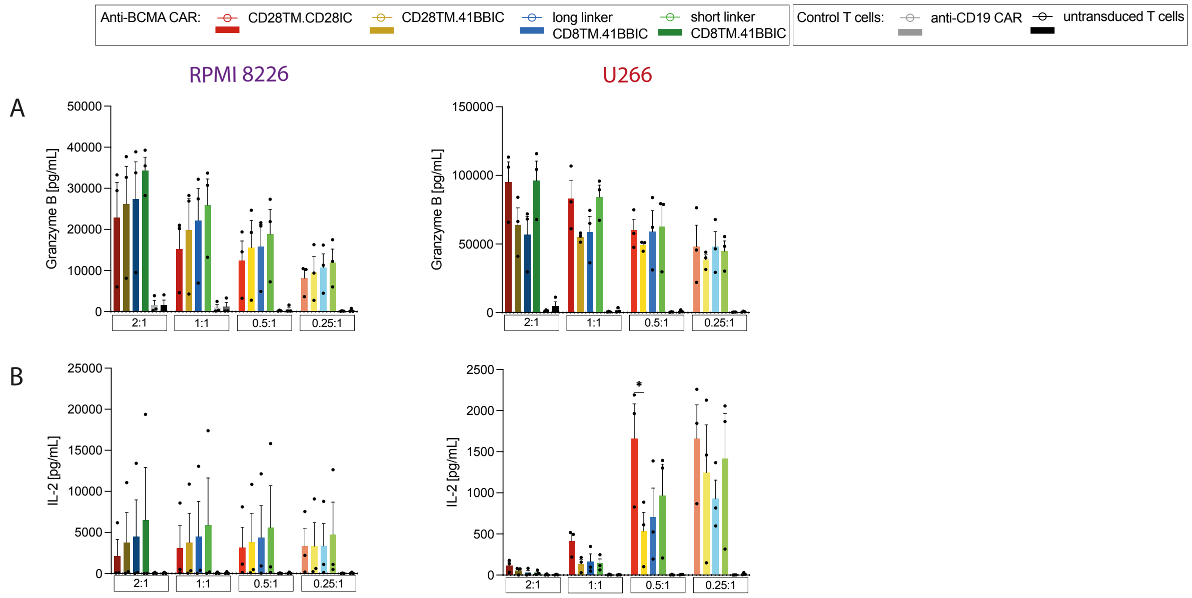

Suppl. Figure 6. Highest Granzyme B production with short linker CD8TM.41BBIC anti-BCMA CAR. **A** Granzyme B and **B** IL-2 ELISA with 72 h co-culture supernatants of anti-BCMA CAR T cells (CD28TM.CD28IC: red; CD28TM.41BBIC: yellow; long linker CD8TM.41BBIC: blue; short linker CD8TM.41BBIC: green), anti-CD19 CAR T cells (gray) and untransduced T cells (black) with RPMI 8226 and U266 for three independent donors (n=3). Each experiment was performed in triplicates. Values in all graphs represent means ± SEM (* $p < 0.05$, ** $p < 0.01$, *** $p < 0.001$, **** $p < 0.0001$). Only selected p-values are shown. Statistical comparison was performed by a 2-way ANOVA with Bonferroni multiple comparison correction.
